# Prevalence, pathogenesis, and clinical impact of pulmonary hypertension associated with chronic obstructive pulmonary disease

**DOI:** 10.3389/fcvm.2026.1700063

**Published:** 2026-05-13

**Authors:** Rodrigo Torres Scabello, Regina Maria de Carvalho-Pinto, Ludmila Neves de Parodi, Gerson de Almeida, Ilma Alves Oliveira Nascimento, Carla Rios da Cruz, Caio Júlio César dos Santos Fernandes

**Affiliations:** Instituto do Coração (InCor), Hospital das Clínicas HCFMUSP, Faculdade de Medicina, Universidade de São Paulo, São Paulo, Brazil

**Keywords:** chronic obstructive pulmonary disease, PH-COPD, phenotype, pulmonary hypertension, targeted therapy, treatment

## Abstract

Pulmonary hypertension (PH) is a progressive condition associated with substantial morbidity and mortality. Chronic obstructive pulmonary disease (COPD), one of the leading global causes of death, frequently coexists with PH, further amplifying functional limitation, increasing exacerbation rates, and elevating mortality risk. Despite its high prevalence, PH-COPD remains under-recognized and lacks evidence-based treatment strategies. This review summarizes current knowledge on its prevalence, pathophysiology, and therapeutic approaches, with particular emphasis on the emerging concept of a “pulmonary vascular phenotype” in COPD. We discuss findings from epidemiological studies, clinical trials of targeted therapies, and registry data, highlighting both inconsistent results and safety concerns. Importantly, we present preliminary real-world data from a Brazilian referral center, which suggests a lower prevalence than previously reported. By integrating global and local perspectives, this review underscores the urgent need for refined phenotyping, biomarker development, and rigorously designed clinical trials to establish therapeutic strategies for this large and vulnerable population.

## Introduction

Pulmonary hypertension (PH) is a hemodynamic condition characterized by an elevation in mean pulmonary arterial pressure (mPAP) >20 mmHg, as assessed by right heart catheterization (RHC). It represents a major global matter, affecting up to 1% of the general population (1). Among elderly groups, the estimated prevalence rises to 10%. Over the past decade, its incidence and prevalence have increased globally; in the United Kingdom, the observed PH prevalence has doubled over the last 10 years (2, 3). PH has a substantial impact on quality of life and survival, with an estimated average survival of approximately 4 years after diagnosis (4).

Diagnostic suspicion should be raised by clinical observation of chronic dyspnea without a confirmed diagnosis or in patients undergoing optimized treatment for other dyspnea-related conditions who fail to achieve the expected outcomes. However, as symptoms are usually non-specific and PH awareness in primary care remains low, challenges persists in its diagnosis and management (5).

Echocardiography is the primary screening tool for PH diagnosis. It provides estimates of systolic pulmonary artery pressure (sPAP), tricuspid regurgitation velocity, and the dimensions of the right ventricle (RV) and right atrium, along with other indirect signs of PH. It can identify pathological conditions of the left heart chambers that may contribute to PH. However, confirmation of PH requires RHC . RHC provides accurate measurements of hemodynamic parameters that corelate with prognosis and enables differentiation between pre-capillary and post-capillary PH, which is essential for PH classification and, therefore, treatment. RHC also allows, when indicated, for vasoreactivity testing and the potential calcium channel blocker therapy (2, 6).

**Figure 1:**
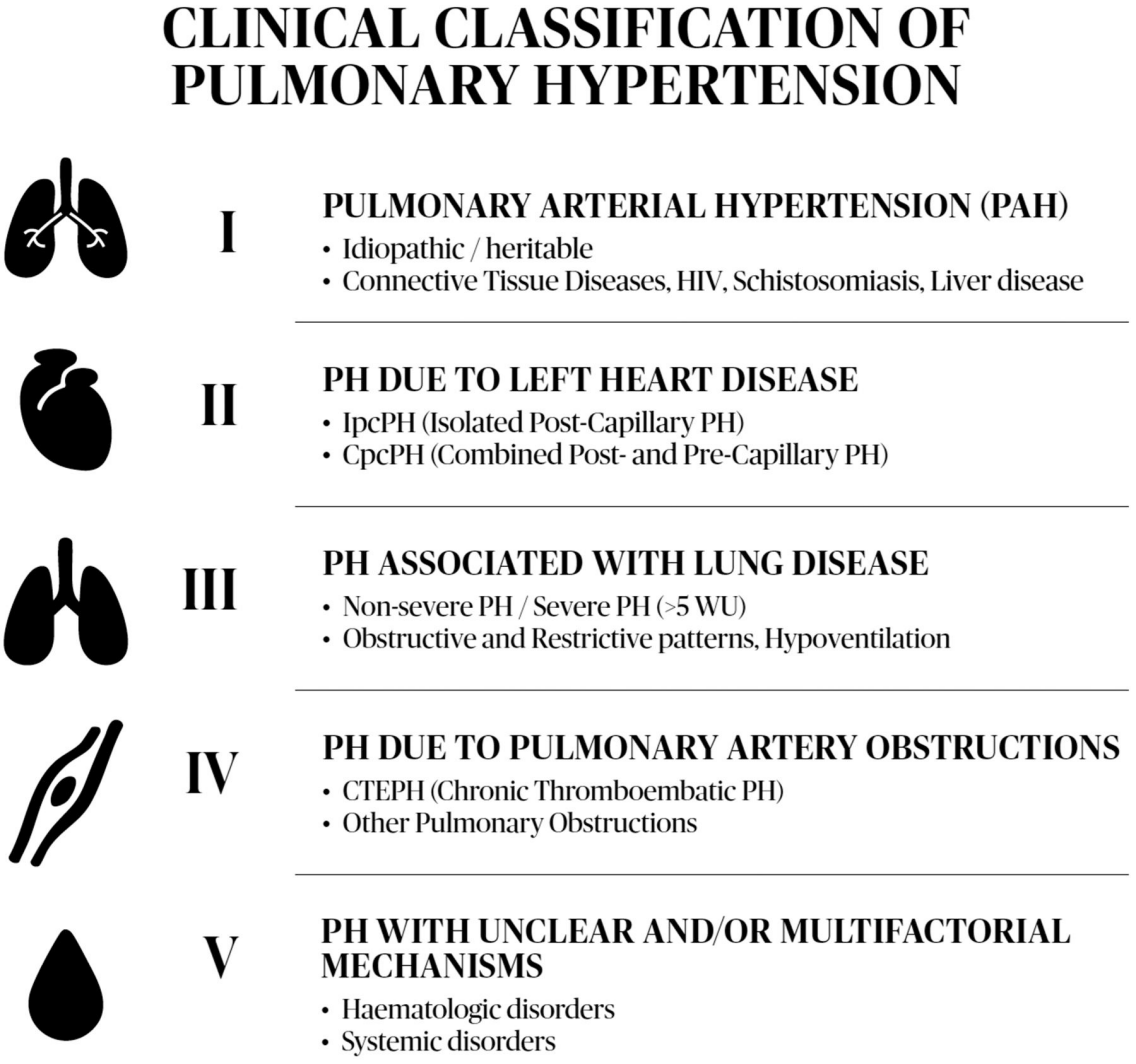
Different PH groups and their etiologies. Adapted with permission from Central illustration by Humbert et al. (© European Society of Cardiology & European Respiratory Society 2026).

The underlying cause of mPAP elevation and potential associated conditions guides the classification of PH. When the pressure increase results from intrinsic changes in pulmonary arterial compliance and endothelial remodeling, the condition is classified as pulmonary arterial hypertension (PAH), or Group I PH, which includes idiopathic, heritable, connective tissue diseases, and drug-induced forms. Patients with left heart disease leading to an increase in hydrostatic pressure, such as heart failure (with preserved, mildly reduced, or reduced ejection fraction) and valvular heart diseases, are classified as Group II PH. Those with chronic thromboembolic pulmonary hypertension are classified as Group IV PH, while patients with unclear and/or multifactorial mechanisms, such as hematological or systemic disorders (e.g., sarcoidosis), are placed in Group V PH (1, 5). However, when elevated pulmonary artery pressure occurs in patients with chronic lung diseases—either restrictive, such as interstitial lung diseases (ILDs), or obstructive, such as chronic obstructive pulmonary disease (COPD)—the condition is classified as Group III PH, which is the focus of this article (2) (see Figure 1).

## Chronic obstructive pulmonary disease

COPD is one of the most prevalent and extensively studied clinical conditions worldwide. Its primary etiology is chronic tobacco smoking, although environmental pollution has gained increasing relevance in recent years. The PLATINO study demonstrated that the prevalence of COPD in Latin America is notably high, particularly among individuals aged 60 years or older, reaching an alarming rate of 25% in this age group. The Burden of Obstructive Lung Disease and other large epidemiological studies estimate a global COPD prevalence of 10.3% among adults (7, 8).

Diagnosis is based on the patient's clinical history, with dyspnea, fatigue, and chronic cough as the main symptoms, in conjunction with exposure to the aforementioned risk factors. Pulmonary function testing via spirometry is essential for diagnostic confirmation. An obstructive pattern is confirmed when the post-bronchodilator ratio of forced expiratory volume in one second (FEV_1_) to forced vital capacity (FVC) is less than 70%, establishing the diagnosis of COPD (7).

**Figure 2:**
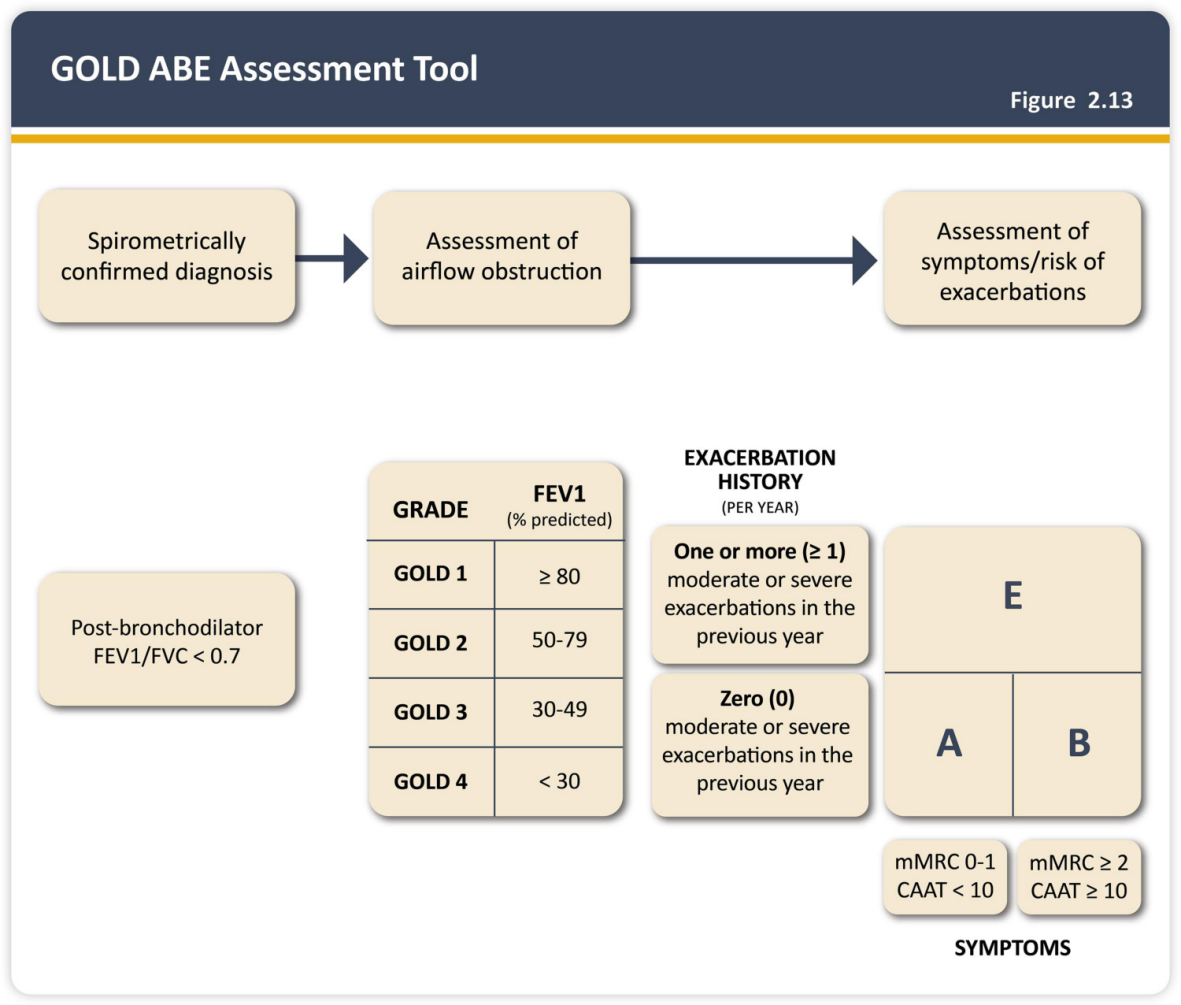
COPD classification by pulmonary function strata (I–IV) and clinical assessment based on symptomatology and exacerbation patterns (ABE) as per the GOLD 2026 guidelines. Reproduced from “2026 GOLD Report” © 2025, 2026 Gold Initiative for Chronic Obstructive Lung Disease, Dear Park, IL, USA.

Spirometric values are also used to classify COPD severity according to the Global Initiative for Chronic Obstructive Lung Disease (GOLD) criteria. Based on post-bronchodilator FEV_1_ values, patients are categorized as shown in Figure 2 (7).

However, spirometric patterns do not always reflect the clinical condition of the patient. Since 2013, GOLD has recommended that classification should also consider clinical symptoms and exacerbation history. A matrix was thus established to jointly assess symptom burden and annual exacerbation frequency. In simplified terms, patients with few symptoms (minimal or no dyspnea) and infrequent exacerbations are classified as Group A. Symptomatic patients (notably dyspnea) without frequent exacerbations are classified as Group B. Patients with one or more severe exacerbations or two or more moderate exacerbations in the past 12 months are considered frequent exacerbators and are classified as Group E (7).

This classification, based on symptomatology and exacerbation patterns, guides therapeutic decisions in COPD, as recommended by GOLD. Patients in Group A may be treated with a single bronchodilator. More symptomatic patients should receive maximal bronchodilation (a long-acting beta-agonist with a combination of a long-acting muscarinic antagonist). Frequent exacerbators should also receive maximal bronchodilation and, when elevated serum eosinophils are present, inhaled corticosteroids may be added (7) (see Figure 2).

## PH related to COPD

**Figure 3:**
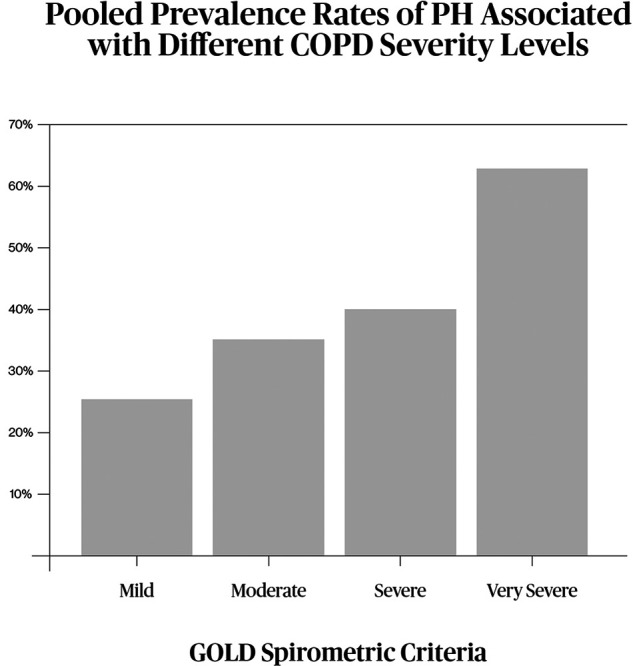
Estimated prevalence of associated PH, stratified by GOLD spirometric criteria, mainly by echocardiographic evaluation in accordance with the metaanalysis by Zhang et al. (10).

Mild to moderate pulmonary hypertension (PH) is a common complication of COPD, and this comorbidity increases the risk of exacerbations, admission costs, and mortality (2, 9). An impacting 39% overall PH prevalence among COPD patients was revealed by the epidemiology meta-analysis by Zhang et al. Stratification by GOLD severity criteria shows a clear association with morbidity progression (Figure 3), with prevalence reaching 61.5% in patients with GOLD stage 4 disease (10). One limitation of these data is that most diagnostics were based on echocardiography rather than RHC, and the study patients were derived from clinical trials, which may not reflect real-world epidemiology.

In our experience at the obstructive pulmonary diseases outpatient clinic of the FMUSP Hospital das Clínicas, we observed lower potential prevalence rates than those reported by Zhang et al. Our preliminary analysis involved a random sample of 202 patients from a larger cohort (mean age 72.8 years; 51.3% male), of whom 96.4% presented with GOLD 2–4 COPD and had undergone at least one registered echocardiographic evaluation. In this sample, 30.7% (*n* = 62) required long-term oxygen therapy (LTOT), and 15.1% had a diagnosis of heart failure. The overall prevalence of PH risk (estimated sPAP >35 mmHg or indirect signs) was 35.1% (*n* = 71/202). Potentially severe PH (estimated sPAP >60 mmHg) (11) was identified in 4.5% (*n* = 9/202) of the sample, assuming that patients with non-measurable tricuspid regurgitation had a low probability of PH.

As potential independent predictors for PH risk, we identified a reduced lung diffusion capacity (DLCO % predicted), where patients with DLCO <50% predicted demonstrated a fourfold increase in the odds of presenting PH (OR: 4.13; 95% CI: 1.28–13.35; *p* = 0.017); the requirement of LTOT, which was associated with an increased prevalence of PH risk (51.6% in the LTOT group vs. 27.9% in non-dependent patients; OR 2.76; *p* = 0.0013); and lower resting ambient air SpO_2_. Those in the PH group presented a lower mean ambient air SpO_2_ of 90.9% compared to 94.2% in the non-PH group (*p* < 0.0001)—an apparently modest difference of 3.3%, although highly statistically significant in this sample.

Notably, we found no statistical correlation between potential PH prevalence and traditional clinical phenotypes (frequent exacerbators profile, GOLD E, compared to non-exacerbators, GOLD A and B: 35.0% vs. 36.6%, respectively; *p* = 0.82) or per the spirometric classification (36.6% for GOLD 3 and 4 vs. 33.3% for GOLD 1 and 2; *p* = 0.628). These initial findings suggest that PH risk might be present across different COPD phenotypes and indicate that impaired gas exchange and hypoxemia *per se* may serve as potential more reliable indicators of pulmonary vascular involvement in COPD than traditional airway-centric parameters. However, these findings remain hypothesis-generating and require further confirmation.

A regression analysis published by Medrek et al. showed that patients with COPD exacerbation-related hospital admissions had a sevenfold higher risk of receiving a PH clinical diagnosis compared to non-hospitalized patients (12). Moreover, there is an established correlation between pulmonary artery enlargement and COPD severe exacerbation risk and mortality (13, 14). The pulmonary artery-to-aorta diameter ratio (PA/Ao) is a sensitive marker for PH prediction and serves as a prognostic tool for COPD and other Group III PH conditions (15). These findings clearly challenge the earlier concept of pulmonary hypertension as a rare condition (16) and point to a more relevant comorbid association between PH and COPD, particularly in patients with more severe conditions.

Although accurate epidemiology estimates of PH-COPD are not yet known, current data indicate that it is one of the leading forms of PH globally (3). Its diagnosis and management could significantly impact millions of patients, particularly those with more advanced disease. COPD itself is a common disease, accounting for 2,512.9 cases per 100,000 individuals (17). Assuming a theoretical PH prevalence of 20%, we would find 502.6 cases per 100,000, substantially higher than the estimated 67.4 PH cases per 100,000 attributed to PH related to left heart disease (Group 2 PH), which is currently considered the most prevalent PH-related form (18). However, a potential confounding factor in these prevalence numbers is that up to 30% of patients with COPD have systolic or diastolic left heart failure; consequently, PH-COPD numbers could be partially inflated by PH cases that are in fact mainly due to left heart disease rather than lung parenchyma alterations (19).

Regarding the different severity grades of PH in COPD, the meaningful clinical impact of mild to moderate PH in COPD patients remains known. In COPD, vascular remodeling may evolve slowly, allowing some patients to develop gradual RV adaptation and remain relatively stable until compensatory mechanisms fail (20). However, mPAP values greater than 35 mm Hg (found in 4%–5% COPD patients) suggest a more complex situation (19). In patients who remain symptomatic despite optimized bronchodilation, it is possible to speculate that a significant proportion of their dyspnea is driven by pulmonary circulatory impairment rather than airway disease alone, meaning that once the right ventricle reaches its physiological limit, even minor further increases in pulmonary vascular resistance (PVR) can precipitate rapid clinical deterioration.

Historically, Chaouat et al. described a subset of COPD patients with relatively preserved lung function but significant dyspnea and fatigue as having “disproportionate” pulmonary hypertension, as the severity of symptoms could not be explained solely by pulmonary function impairment but was attributed to circulatory limitation (impaired cardiac output augmentation) (21). However, recent consensus (20, 22) recommends defining this subgroup as the “pulmonary vascular COPD phenotype.” In these cases, vascular lesions are typically morphologically similar to those observed in idiopathic pulmonary arterial hypertension and are characterized by severe hemodynamic impairment with relatively mild pronounced ventilatory limitation, suggesting that vascular proliferative pathogenic mechanisms may be superimposed on the underlying COPD (20, 22).

The current definition of severe PH-COPD, a high-mortality phenotype, is based on pulmonary vascular resistance, as proposed by Zeder et al. (2, 23). The authors identified that a PVR >5 Wood Units (WU) had the strongest predictive value for mortality in patients with moderate COPD. A later study by Piccari et al. confirmed the prognostic significance of this threshold in a more heterogeneous COPD population (24). Frequently, these patients with elevated PVR also meet an older severity criterion, defined by a mPAP ≥35 mmHg (2).

Boerrigter et al. demonstrated that patients with COPD and severe PH exhibit a predominantly circulatory limitation during exercise —characterized by the inability of the cardiovascular system to sufficiently increase oxygen delivery to meet tissue demands during exertion—rather than the ventilatory limitation typically associated with the disease. This cardiocirculatory limitation is reflected by exhaustion of the oxygen reserve, a reduced stroke volume index, and a decreased slope of the cardiac output-to-oxygen consumption (CO/VO_2_) curve during maximal exercise. Notably, this same group did not exhibit ventilatory limitation, maintaining a significant breathing reserve (37% ± 11%) and showing no evidence of hypercapnia (PaCO_2_ accumulation), which is commonly observed in other COPD patients. Clinically, this profile translates into a severely compromised exercise capacity, reflected by a significantly lower 6-min walk distance (6MWD) (208 ± 144 m). These findings suggest that benefits of pulmonary vasodilator therapy would likely be restricted to this specific pulmonary vascular phenotype (25).

## PH-COPD pathophysiology

### Hypoxia and endothelial dysfunction

The main cause of the potential rise in pulmonary arterial pressure in COPD is pulmonary vascular remodeling, likely resulting from the combined effects of hypoxia, inflammation, and capillary loss in advanced emphysema (1, 2, 21). Chronic alveolar hypoxia, a cornerstone of severe COPD, triggers acute vasoconstriction as an adaptive mechanism to optimize ventilation/perfusion (V/Q) matching. However, persistent hypoxia leads to endothelial dysfunction, characterized by an imbalance between vasodilators (nitric oxide, prostacyclin) and vasoconstrictors (endothelin-1). This biochemical shift promotes the proliferation of smooth muscle cells and fibroblasts, leading to muscularization of non-muscularized distal arterioles and intimal thickening. These structural changes result in a permanent increase in pulmonary vascular resistance, effectively “fixing” the hypertension beyond the initial phase of functional vasoconstriction.

At the molecular level, sustained hypoxia activates hypoxia-inducible factor signaling, increases endothelin-1 activity, and reduces nitric oxide bioavailability, thereby promoting vasoconstriction, smooth muscle proliferation, and extracellular matrix deposition. Over time, these changes shift the pulmonary circulation from a predominantly functional, vasoconstrictive state to a fixed structural increase in pulmonary vascular resistance. This transition is clinically relevant, as it helps explain why some patients exhibit limited reversibility and why indiscriminate vasodilation may aggravate V/Q mismatch by increasing perfusion to poorly ventilated regions.

### Inflammation and immune dysregulation

**Figure 4:**
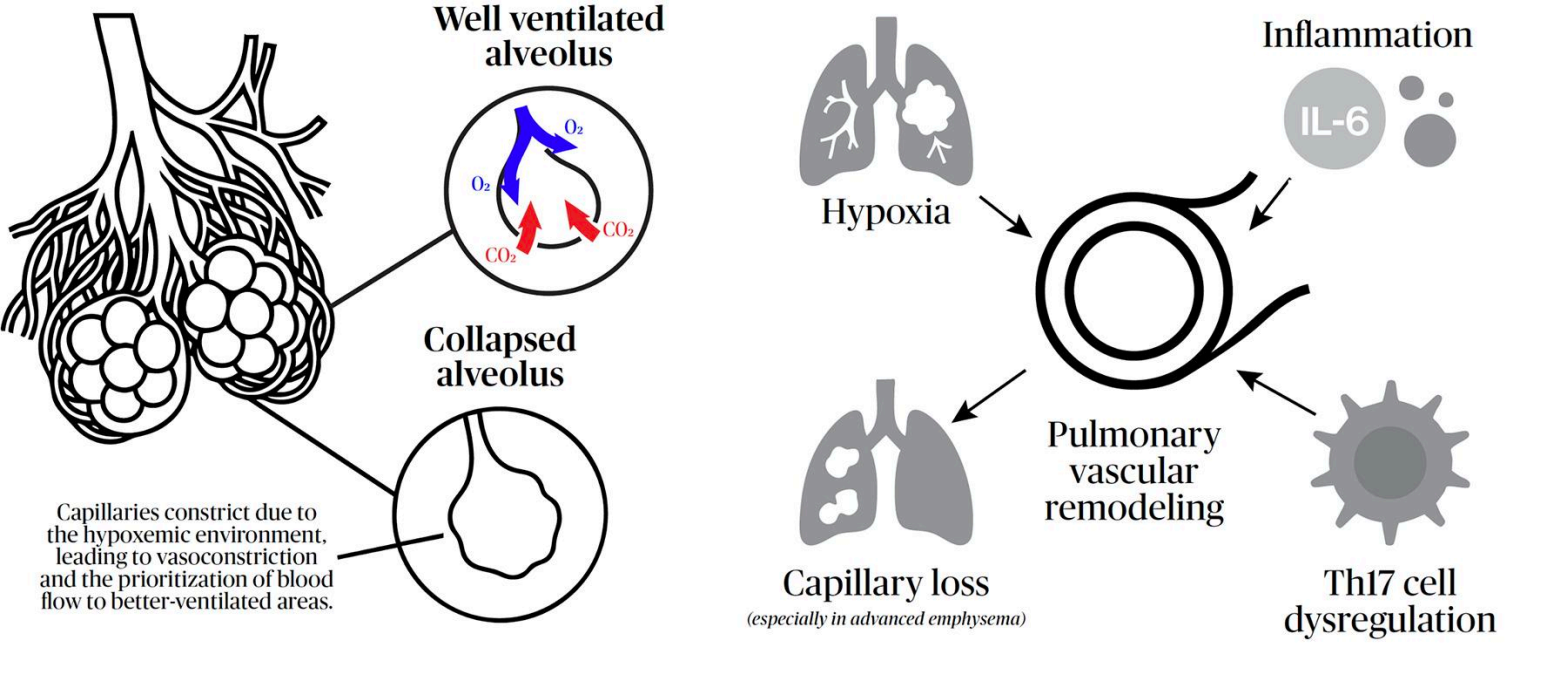
Schematic representation of the main mechanisms linking COPD to pulmonary hypertension, integrating hypoxic vasoconstriction, endothelial dysfunction, inflammatory signaling, and structural vascular remodeling. The figure also illustrates how increased pulmonary vascular resistance leads to progressive right ventricular afterload and maladaptive remodeling. These pathways help explain both the heterogeneity of PH severity among COPD patients and the variable physiological response to pulmonary vasodilator therapies.

Beyond hypoxia *per se*, recent evidence highlights the role of chronic systemic and local inflammation in pulmonary vascular impairment. Pro-inflammatory cytokines, mainly IL-6, along with immune cell infiltration (macrophages and T-lymphocytes) and dysregulation of T helper type 17 (Th17) responses (26, 27) (see Figure 4), contribute to a pro-proliferative environment within the vessel wall. This immunopathogenetic component might explain why some patients develop significant PH despite relatively preserved lung volumes, as inflammatory “spillover” from the airways to the vasculature can initiate remodeling independently of the degree of bronchial obstruction. However, this remains an area requiring further research to better elucidate the immunopathogenic mechanisms.

### Right ventricular adaptation and cor pulmonale

The right ventricle (RV) is the ultimate determinant of clinical outcomes and prognosis in the pathology of PH-COPD. Challenged by a rise in pulmonary artery pressure, the RV undergoes adaptive hypertrophy to maintain stroke volume against increased afterload. However, due to its thin-walled structure, the RV is not suited to prolonged high-pressure states, leading to chamber dilation and contractile failure, a clinical condition termed cor pulmonale. In addition to this hemodynamic hurdle, lung hyperinflation increases intrathoracic pressure and further impairs RV filling. Understanding this RV–pulmonary vascular coupling is crucial, as therapeutic interventions that inadvertently worsen V/Q mismatch by inhibiting hypoxic pulmonary vasoconstriction may further impact the pulmonary circulation by increasing its workload without improving systemic oxygenation (28, 29). Accordingly, RV dysfunction is a major determinant of exercise intolerance, peripheral congestion, hospitalization risk, and mortality in PH-COPD, underscoring that “cor pulmonale” is not merely a historical term but a clinically meaningful phenotype within Group 3 PH.

## General lines of pharmacological treatment for PH

Three main therapeutic pathways are well established in PH treatment: the prostacyclin, endothelin, and nitric oxide pathways. All aim to reduce pulmonary vascular pressure gradients through direct and indirect vasodilation mechanisms (2, 27, 30). Recently, a new therapeutic pathway has emerged, particularly in PAH: the activin pathway. This pathway, along with the drug sotatercept, is a therapeutic approach aimed at reducing intima proliferation and has demonstrated impactful effects on vascular remodeling. To our knowledge, no current clinical study has evaluated this pathway in PH-COPD (31).

One might question the rationale for using inhaled drugs in PH, as the disease primarily affects the vasculature rather than the airways. However, poorly ventilated regions are usually also poorly perfused. Therefore, delivering inhaled drugs to better-ventilated regions is advantageous for two main reasons: reduction in vascular resistance is more effective in regions with higher blood flow. Second, this approach avoids vasodilation in poorly ventilated and perfused areas, which could cause pulmonary shunting and worsening hypoxemia. Since inhaled particles preferentially reach well-ventilated areas, this strategy targets the desired segments while avoiding vasodilation in non-ventilated regions, potentially preventing true shunting and secondary hypoxemia. On the other hand, it is acknowledged that inhaled medications eventually reach the systemic circulation. Although the systemic transition of these drugs is a known pharmacokineticphenomenon, the clinical benefits observed in studies, such as those evaluating inhaled treprostinil in Group 3 PH (including ILD), are largely attributed to this potential preferential local–regional action, which preserves gas exchange stability (19).

In summary, the following clinical trials, including inhaled, subcutaneous, and oral medications, were performed in PH-COPD patients.

### Prostacyclin pathway

The PERFECT trial, a phase 3 randomized controlled trial (RCT) evaluating inhaled treprostinil in PH-COPD, was terminated early due to an increased risk of serious adverse events and mortality. Clinically, patients receiving treprostinil also showed worse performance in the 6MWT than those in the placebo group (32). Interestingly, a *post hoc* analysis demonstrated that a subgroup of patients with sPAP >40 mmHg and FEV_1_ > 40% presented clinical benefit with the therapy (33).

An earlier open-label study by Bajwa et al. evaluating inhaled treprostinil also showed no improvement in functional outcomes and reported declines in FEV_1_, FVC, and diffusing capacity (DLCO); however, the study included nine patients, below the initial minimum planned sample size of 20 (34).

Abuserewa et al. performed a retrospective case series involving six patients treated with selexipag for 6 months and presented improvements in 6MWD (+97 m), mPAP (−10 mmHg), and PVR (−4.7 WU); however, no formal statistical analysis was performed (35).

### Endothelin pathway

Two trials evaluating bosentan in PH-COPD have yielded conflicting results. A study by Stolz et al. in patients with non-severe disease showed no benefits and, instead, reported worsening hypoxemia, reduced quality of life, and poor tolerability (36). In contrast, an open-label trial by Valerio et al. in patients with severe PH-COPD reported significant improvements in exercise capacity (+71 m 6MWD) and PVR (37). However, the non-blinded design of the latter study warrants a cautious interpretation of its positive findings (38). The ARIES-3 study, an open-label trial (2012) evaluating ambrisentan across different PH etiologies, included 24 COPD patients. This subgroup showed reduced BNP levels but no improvement in 6MWD (39). Overall, ERAs have shown inconsistent results and raised safety concerns (38).

### Nitric oxide pathway

Several RCTs have evaluated sildenafil and tadalafil in PH-COPD and yielded mixed results. The SPHERIC-1 trial by Vitulo et al. was the only RCT that used RHC to confirm severe PH. This study showed significant improvements in PVR, cardiac index (CI), BODE index, and DLCO, but no significant change in 6MWD (40). Rao et al. found a 190 m increase in 6MWD and a reduction in sPAP with sildenafil (41). In contrast, Blanco et al.(42), in a trial involving 63 patients, and Goudie et al. (43), studying 120 patients, found no improvement in exercise capacity or quality of life with sildenafil and tadalafil, respectively. Moreover, a recent RCT by Maron et al. involving 42 patients showed that tadalafil reduced dyspnea and exacerbations but did not improve 6MWD or survival (44).

Table 1 provides an overview of the principal clinical studies conducted in PH-COPD, highlighting the heterogeneity of inclusion criteria, inconsistency of reported outcomes, and methodological limitations. These factors collectively explain why current evidence remains insufficient to support firm recommendations in contemporary guidelines.

**Table 1:** Clinical studies of targeted therapies in PH-COPD.

Therapeutic pathway/drug	Study (year)	Design/*N*	Inclusion criteria	Follow-up duration	Main results	Limitations
Prostacyclin—Inhaled treprostinil	PERFECT (2024)	Phase 3 RCT, cross-over study	PH-COPD confirmed	12 weeks (terminated early)	↑ Adverse events, ↓ 6MWD	Early termination, small *N*, heterogeneous population
	PERFECT—post hoc	Subgroup, cross-over study	sPAP >40 mmHg, FEV1 > 40%	12 weeks (terminated early)	Suggestion of benefit	Exploratory, non-planned
	Bajwa et al. (2017)	Open-label, *N* = 9	PH-COPD	16 weeks	No functional improvement; ↓ FEV1/FVC/DLCO	Very small sample
Prostacyclin—Selexipag	Abuserewa et al. (2021)	Case series, *N* = 6	“Out-of-proportion” PH-COPD	6 ± 2 months	+97 m 6MWD, ↓ mPAP (–10 mmHg), ↓ PVR (–4.7 WU)	No control group, descriptive only
ERA—Bosentan	Stolz et al. (2008)	RCT, *N*≈30	COPD, non-severe PH	12 weeks	No benefit; worsened hypoxemia/QoL	PH not severe, tolerability issues
	Valerio et al. (2009)	Open-label, *N*≈30	Severe PH-COPD	18 months	+71 m 6MWD, ↓ PVR	Open, non-controlled
ERA—Ambrisentan	Badesch et al./ARIES-3 (2012)	Open-label, subgroup COPD (*N* = 24)	Mixed PH etiologies	24 weeks	↓ BNP; no 6MWD improvement	Small subgroup, non-specific
PDE5i—Sildenafil	Rao et al. (2011)	RCT, *N*≈30	COPD + PH	12 weeks	+190 m 6MWD, ↓ sPAP	Small study, variable criteria
	Blanco et al. (2013)	RCT, *N* = 63	Pulmonary rehab ± sildenafil	3 months	No 6MWD/QoL benefit	Negative trial
	SPHERIC-1/Vitulo (2016)	RCT, *N*≈28	Severe PH-COPD, RHC confirmed	16 weeks	↓ PVR, ↑ CI/DLCO/BODE; no 6MWD change	Small, functional endpoint negative
PDE5i—Tadalafil	Goudie et al. (2014)	RCT, *N* = 120	Moderate-to-severe COPD; PH evaluation by Echocardiography	12 weeks	No 6MWD/QoL benefit	Large, but negative
	Maron et al. (2022)	RCT, *N* = 42	Veterans with PH-COPD	12 months	↓ Dyspnea, ↓ exacerbations; no 6MWD/survival benefit	Small, short follow-up
sGC stimulators—MK-5475	INSIGNA-PH-COPD (ongoing)	Phase 2 RCT	COPD + PH	24 weeks	Inhaled sGC stimulator	Ongoing
PDE5i—Tadalafil	ERASE (ongoing)	Phase 2 RCT	Severe PH-COPD	16 weeks	Efficacy/safety endpoints	Ongoing

ERA, endothelin receptor antagonist; PDE5i, phosphodiesterase-5 inhibitor; sGC, soluble guanylate cyclase; QoL, quality of life; BODE index, Body mass index, airflow Obstruction, Dyspnea, and Exercise capacity index. In all trials, PH diagnostic confirmation was performed by RHC, except for the Goudie et al. (43) trial, as indicated in the table.

Taken together, these findings highlight not only the paucity of robust evidence in PH-COPD but also the likelihood that therapeutic responses are confined to a distinct vascular phenotype. This underscores the urgent need for future trials to incorporate refined phenotyping strategies and hemodynamic stratification to identify patients most likely to benefit from targeted therapies.

## Status of evidence and future directions

The current evidence base for PH-COPD remains fragmented, with most available studies limited by small sample sizes, heterogeneous inclusion criteria, and short follow-up durations. Functional outcomes, such as 6-min walk distance, have shown inconsistent results, even in the presence of hemodynamic improvements. This discrepancy suggests that the benefits of targeted therapies may be confined to a distinct “vascular phenotype”—patients with disproportionate pulmonary hypertension who exhibit marked hemodynamic impairment despite relatively preserved ventilatory function. Identifying this subgroup is critical, as it may represent the population most likely to derive clinical benefit. To address these gaps, ongoing phase II trials, such as ERASE (tadalafil) and INSIGNA-PH-COPD (inhaled sGC stimulator) are now explicitly incorporating hemodynamic stratification and more rigorous endpoints, which may help clarify the therapeutic role of targeted drugs in this challenging condition.

Several critical knowledge gaps remain. First, the true epidemiological burden of PH-COPD is still uncertain, largely because most prevalence estimates rely on echocardiographic surrogates rather than right heart catheterization. Second, the natural history of mild to moderate PH in COPD is poorly characterized, and its independent contribution to morbidity and mortality is not fully understood. Third, there is a lack of validated biomarkers or imaging markers capable of reliably distinguishing patients with a predominant vascular phenotype from those whose symptoms are primarily driven by ventilatory limitation. Addressing these gaps will require large, multicenter prospective studies specifically designed to capture hemodynamic severity, longitudinal outcomes, and patient-reported measures of quality of life.

Beyond research priorities, the clinical and policy implications are equally pressing. At present, the management of PH-COPD relies almost exclusively on optimization of COPD care, long-term oxygen therapy, and supportive measures, leaving patients with severe hemodynamic compromise as therapeutic orphans. Strengthening referral networks, increasing awareness of PH in COPD patients who remain symptomatic despite optimized treatment, and expanding access to specialized centers with right heart catheterization capabilities are key immediate steps. In parallel, health policy should prioritize the inclusion of PH-COPD in educational initiatives, patient registries, and clinical trial infrastructures. Only through such a dual approach— combining rigorous scientific inquiry with pragmatic health system strategies—can we move closer to delivering effective, evidence-based care for this highly vulnerable population.

Our group in Brazil is developing a phase II clinical trial—HOLLYWOOD—to investigate the effects of 12-week inhaled iloprost therapy in a subset of PH-COPD with severe hemodynamic parameters. Results are expected in the second half of 2026.

## Conclusions

PH-COPD represents one of the most prevalent and clinically relevant forms of pulmonary hypertension worldwide. Although observational data and small interventional studies have provided important insights, the evidence remains inconsistent, and no therapy is currently established as a standard of care. The recognition of a vascular phenotype in COPD—characterized by disproportionate hemodynamic impairment and poor outcomes—may allow for more precise identification of patients who could benefit from targeted interventions. Our analysis also suggests that epidemiological estimates may be inflated by overlapping cardiac disease, reinforcing the need for careful phenotyping.

Future research should prioritize:

1. Rigorous prevalence studies using right heart catheterization;
2. Clinical trials stratified by hemodynamic severity and phenotype;
3. The incorporation of patient-centered outcomes, such as quality of life and functional capacity;
4. Policies aimed at increasing awareness and enhancing diagnostic capacity in primary and secondary care settings.

Until such advances are made, PH-COPD remains a therapeutic orphan condition, with management relying mainly on optimized COPD care, long-term oxygen therapy, and individualized referral to specialized centers.
